# The Potential of Virtual Reality for Inducing Neuroplasticity in Children with Amblyopia

**DOI:** 10.1155/2020/7067846

**Published:** 2020-06-29

**Authors:** María B. Coco-Martin, David P. Piñero, Luis Leal-Vega, Carlos J. Hernández-Rodríguez, Joaquin Adiego, Ainhoa Molina-Martín, Dolores de Fez, Juan F. Arenillas

**Affiliations:** ^1^Group of Applied Clinical Neurosciences and Advanced Data Analysis, Neurology Department, Faculty of Medicine, University of Valladolid, Valladolid, Spain; ^2^Department of Optics, Pharmacology and Anatomy, University of Alicante, Alicante, Spain; ^3^Department of Ophthalmology, Vithas Medimar International Hospital, Alicante, Spain; ^4^Group of Applied Clinical Neurosciences and Advanced Data Analysis, Computer Science Department, School of Computing, University of Valladolid, Valladolid, Spain; ^5^Department of Neurology, Stroke Unit and Stroke Program, University Hospital, University of Valladolid, Valladolid, Spain

## Abstract

In recent years, virtual reality (VR) has emerged as a new safe and effective tool for neurorehabilitation of different childhood and adulthood conditions. VR-based therapies can induce cortical reorganization and promote the activation of different neuronal connections over a wide range of ages, leading to contrasted improvements in motor and functional skills. The use of VR for the visual rehabilitation in amblyopia has been investigated in the last years, with the potential of using serious games combining perceptual learning and dichoptic stimulation. This combination of technologies allows the clinician to measure, treat, and control changes in interocular suppression, which is one of the factors leading to cortical alterations in amblyopia. Several clinical researches on this issue have been conducted, showing the potential of promoting visual acuity, contrast sensitivity, and stereopsis improvement. Indeed, several systems have been evaluated for amblyopia treatment including the use of different commercially available types of head mounted displays (HMDs). These HMDs are mostly well tolerated by patients during short exposures and do not cause significant long-term side effects, although their use has been occasionally associated with some visual discomfort and other complications in certain types of subjects. More studies are needed to confirm these promising therapies in controlled randomized clinical trials, with special emphasis on the definition of the most adequate planning for obtaining an effective recovery of the visual and binocular function.

## 1. Introduction

Among the more recent products resulting from the evolution of digital technology, virtual reality (VR) has become increasingly entrenched in the field of clinical medicine, emerging as a new safe and effective tool in particular with regard to neurorehabilitation of different childhood and adulthood conditions [1–5]. Consistent in the presentation of computer-generated 3D environments, VR enables users to become fully immersed in a simulated world in which they can interact via multiple sensory channels: visual, auditory, or haptic [2].

Professionals also benefit from this technique, as they can have a high degree of control over the whole therapeutic experience of the patient. Likewise, the compatibility of VR with high-precision functional imaging techniques, such as functional magnetic resonance imaging (fMRI), represents an advantage for researchers, as it allows them to present multimodal stimuli with a high degree of validity and ecological control while recording changes in subject's brain activity [6].

So far, VR-based therapies have been shown to induce cortical reorganization and promote the activation of different neuronal connections over a wide range of ages [7–10], leading to contrasted improvements in some motor and functional skills, such as gait or balance [11, 12], while their potential contributions to the visual system remain virtually unexplored. Actually, most authors who have considered the study of VR interventions in relation to vision have focused their efforts on describing the short- and long-term ocular side effects associated with its use and on recreating different performance tests to try to identify common eye disorders or related impairments [13–16], with only a few who have attempted to use this tool to improve visual abilities that are not being adequately addressed by existing treatment options [17–20].

In this context, the use of VR technology as a potential treatment for improving vision in amblyopia is recently attracting considerable interest because of the possibility of training each eye independently without the need of occlusion or penalization. This dichoptic stimulation approach has the potential of eliminating one of the major causes of amblyopia treatment failure especially among the pediatric population, which is poor compliance to patching due to social stigma or long treatment duration or the physical properties of the patch itself (design, heat, irritation, poor adhesive material, etc.) [21].

Therefore, the purpose of this article is to review all the research available in the scientific literature on the use of VR-based interventions for the treatment of amblyopia in children, as well as compiling evidence-based recommendations to properly guide the conduct of future studies, given that most of the randomised clinical trials (RCTs) published to date have been found to be not free from methodological issues [22].

## 2. Supporting Evidence

According to Hebbian theory, robust, synchronous activation in neural populations leads to enhanced synaptic strength between the neurons comprising those populations [23], and like electrical or magnetic stimulation of brain areas, visual feedback can induce such neuroplastic changes in sensory-motor networks.

This means that VR technology can be used to deliver meaningful and relevant stimulation to the brain and thereby trigger neuroplasticity mechanisms to promote the rehabilitation of sensory-motor deficits given in different disorders.

To this end, visual stimuli can be presented to the user in two ways: on a monitor screen or in a fully immersive environment generated through the use of technological equipment, such as head mounted displays (HMDs) and motion capture systems.

Over the last years, the gaming industry is particularly taking advantage of these developments in a field where interaction has been traditionally two-dimensional and nonnatural (either with a simple mouse or with game controllers), since HMDs provide stereoscopic images for fully immersing experiences and motion capture systems allow the creation of visual-motor correlations between the user's movements and those of an avatar (virtual representation of the user) [24].

Furthermore, these technologies are mostly well tolerated by users during short time exposures and do not cause significant long-term side effects, although it should be noted that the use of HMDs has been occasionally associated with problems, ranging from the mildest including visual disturbances, dizziness, and nausea [25, 26] to seizures and epileptic spams in photosensitive subjects, especially in children [27]. Some authors pointed out the vergence-accommodation conflict as responsible of the visual discomfort associated with the use of VR HMDs [28], which appears to be more likely to occur at closer viewing distances [29]. Likewise, neurological events can also occur with this therapy as a result of high sensitivity to overexcitation in some cortical regions of the subject, as has been also reported with other neuromodulation techniques, developed to improve visual function in amblyopia, such as transcranial Direct Current Stimulation (tDCS) [30–33] or repetitive Transcranial Magnetic Stimulation (rTMS) [34, 35]. All these issues should be considered when designing future clinical studies that intend to use VR HMDs as stereoscopic tools with rehabilitation aims.

The main feature of VR games adapted for healthcare purposes, often called serious games (SGs), is their inherent capacity to engage players at multiple levels (cognitive, physical, perceptual, etc.) and motivate them to repeat gameplay [36, 37], which in the particular case of amblyopic children seems to be a great advantage, considering the existing poor compliance of these patients with conventional therapy.

Previous cross-sectional studies have shown that children who play SGs exhibit increased cortical thickness as well as a greater regional volume of grey matter in the dorsolateral prefrontal cortex (PFC), hippocampal formation, frontal eye fields, insula, and cerebellum [38–40], which contribute to the enhancement of a range of cognitive skills including attention, processing of information, task switching, and mental rotation [41–45].

Over the time, a relationship between SGs practice and improvement in certain types of visual cognition abilities has been also well established [46]. For instance, it has been shown that playing action SGs is linked to enhanced peripheral vision [47], superior spatial skills [48], improved contrast sensitivity [49], and reduced crowding effect [50], among others benefits.

In addition, recent research provides examples of how the presentation of visual stimuli that can be integrated into the dynamics of different SGs could result in better clinical performance for a variety of visual conditions such as myopia, presbyopia, age-related macular degeneration (AMD), and, of course, amblyopia [51–53].

Considering all these lines of evidence, this article adds to a short history of reviews into ways in which sensory training through VR-based video-game experiences can improve visual abilities in children suffering from amblyopia.

## 3. Previous Research of the Use of VR on Amblyopia

In multiple sensory skills, including vision, performance in simple and basic tasks can be improved with extensive practice and training [54]. This treatment modality, known as perceptual learning, can be implemented into the dynamics of different SGs available for distinct VR interfaces and has shown to be a useful therapy approach for enhancing visual impairments that occur in amblyopia even after the critical period of visual development [55]. In fact, a review conducted by Levi and Li in 2009 proved that visual improvements produced by 20 hours of perceptual learning in amblyopic older children and adults were equivalent to those derived from about 500 hours of patching [56].

Particularly in young children, perceptual learning via playing SGs has led to similar results. For example, a pilot clinical study conducted by Polat et al. in 2009 found that playing two sessions of SGs per week (with a total number of no more than 40 sessions and a practice time of approximately 30 minutes each) increased visual acuity by 1.5 Snellen lines (or 2.12 ETDRS lines) and restored contrast sensitivity function to the normal range in amblyopic children aged 7 to 8 years who were noncompliant with patching or in whom patching had failed despite good compliance [57].

The method followed by other authors was to combine this perceptual learning approach with dichoptic training, which consist in viewing a separate and independent field by each eye with increased stimulation to the amblyopic [58–61]. On this field, Li and colleagues demonstrated that 9 hours of visualizing dichoptic movies over a period of 2 weeks resulted in 1 to 4 lines of visual acuity improvement in amblyopic children aged 4 to 10 years [62], while patching by comparison requires 120 hours of treatment to achieve 1 line of visual acuity improvement in children with amblyopia who have already been treated with spectacles for a period over 12–16 weeks [63].

Since the recent boom of VR technology, some attempts have been made to see if the visual improvements obtained with these therapies can be enhanced by integrating them into a virtual and interactive environment (Table 1). Some of the first authors to explore this possibility were the I-BiT system group, who developed an interactive VR-based binocular system to treat amblyopia via playing interactive computer games or viewing 3D DVD footage with 3D shutter glasses. This system used specially configured software to preferentially stimulate the amblyopic eye without compromising the vision of the unaffected (dichoptic stimulation), and its potential to improve visual acuity in amblyopia has been proved in different age ranges both when the therapy was applied in isolation [64–66] and in combination with patching [67]. To assess the efficacy of the system in a larger population, RCT was conducted with a sample of 75 amblyopic children aged 4 to 8 years [68], in which the visual acuity of the amblyopic eye improved by approximately 0.07 logMAR at 6 weeks of treatment in all therapy arms. However, no statistical significance was observed between viewing I-BiT DVDs or playing non-I-BiT games compared with playing I-BiT games (stated primary outcome) in terms of gain in vision. On the other hand, the treatment did not induce any adverse effects among the participants, so dichoptic stimulation using 3D shutter glasses technology can be considered a safe alternative to provide immersive sensory feedback to amblyopic patients.

Similarly, Qiu and colleagues proposed a full-field vision system aimed to provide interactive VR computer games for binocular treatment of amblyopia, the Viston-VR™ system [71]. The main feature of this system is using two dissociated optical systems to provide independent displaying contents for each eye, so each eye can be stimulated by viewing different cartoon films or playing interactive VR games by means of specially devised glasses. In the initial evaluation of this system, the patient received a weekly treatment which consisted of watching cartoon films for 10 minutes and playing VR games for 20 minutes daily. The preliminary clinical results obtained at the Second Affiliated Hospital Zhejiang University School of Medicine showed an obvious effect of the therapy on improving patients' visual acuity function, shortening the treatment period compared to the traditional treatment. In summary, the therapy proved to be safe and well tolerated for all patients, although the sample size used in the study was not specified.

Rastegarpour described in 2011 another anaglyphic VR-based system with the potential of being an option for amblyopia management [72]. The prototype of the system, the ABG InSight, was found capable of successfully filtering out elements of a certain color and therefore could prove to be a viable alternative to existing VR-based systems for treating amblyopia. In this case, visual stimuli are presented to the patient by means of a pair of glasses with two color filters and software for use on a personal computer. Using this system, which has not been clinically validated yet, VR-based training could be performed forcing the patient to use both eyes, specifically the amblyopic eye, while playing computer games. Likewise, the option of using a dichoptic global motion technique to measure interocular suppression in children with amblyopia has been also explored [73]. This technique consisted of presenting high contrast signal dots to the amblyopic eye, while maintaining the presentation of low contrast noise dots to the nonamblyopic fellow eye. During the measurement procedure, the contrast of the noise dots was increased until discrimination of the motion direction of the signal dots reached chance performance. With this methodology, the authors were able to demonstrate that the interocular suppression was stronger in strabismic amblyopia than in anisometropic amblyopia. This methodology has been subsequently used by other authors with some modifications [74].

Besides all this scientific background on the use of VR in amblyopia, additional studies have been conducted in the last few years to evaluate new VR-based prototypes or approaches for its treatment. Cepeda-Zapata et al. [69] proposed a system for performing conventional visual therapy exercises (Brock cord, approach technique, or convergence-divergence stimulation) into VR environments, validating it in a sample of 45 students who confirmed its simplicity and versatility. In the same way, in 2017, a pilot study was conducted to evaluate the effectiveness of dichoptic visual training (Diplopia Game, Vivid Vision) using a VR HMD (Oculus Rift OC DK2) in a sample of 17 anisometropic amblyopic adults [70]. Visual acuity and stereopsis changes were evaluated after eight sessions of 40 minutes each, showing a significant visual improvement and change in stereopsis. Specifically, a total of eight patients (47.1%) before dichoptic treatment had unmeasurable stereoacuity, while this only occurred in two patients (11.8%) after training. Occasionally, some patients felt tired after the use of the immersive device and reported a sensation of pulling behind the amblyopic eye that subsided over time, without major side effects.

There are also nonimmersive VR systems that have shown a potential to improve cognitive and motor abilities, even in advanced stages of several neurological disorders [75–77]. These systems that allow interaction with the environment via mouse or joystick are cheaper and easier to be understood by users [78]. Unfortunately, no scientific articles of quality investigating the effect of such systems in amblyopic patients have been reported to this date.

According to all these lines of evidence, VR dichoptic and perceptual learning training seem to be a useful therapeutic option for achieving a successful visual rehabilitation in amblyopic patients. Furthermore, these systems allow clinicians to measure and control changes in interocular suppression, which is considered one the main factors leading to cortical alterations in amblyopia [79]. More clinical studies are needed to confirm the promising results of these therapies in RCTs, with special emphasis on the definition of the most adequate planning for obtaining an effective recovery of visual and binocular functions. Likewise, it is necessary to conduct RCTs to evaluate the potential benefit of VR-based training in amblyopia over conventional approaches or perceptual learning or dichoptic training in non-VR environments. Furthermore, future clinical studies should evaluate the impact of VR-based treatments for amblyopia in larger samples of patients. Finally, a more detailed and scientific-based definition of the stimuli used in VR-based trainings in amblyopia should be performed in order to optimize this type of treatment.

## 4. Methodological Framework for the Development of Future Studies on VR

Several researchers have pointed out four key factors to consider about VR and its application for neurorehabilitation in clinical studies:

1. Repetition: neuroplasticity is use-dependent and intensive and repetitive training is therefore crucial to enhance the promotion of functional changes in the neural architecture. In this context, adaptive training paradigms that continually move the subject's performance towards the targeted skill are considered necessary to optimize the performance of that skill [80]. In this context, VR-based therapies can be used to provide learning algorithms and/or rehabilitation activities that can be methodically manipulated to meet this need.
2. Sensory feedback: scientific evidence indicates that maximum development of neural networks can only be obtained by working through different channels, so multisensory stimulation is considered another essential component for inducing brain restructuring. Versatility of VR in presenting complex sensory stimulation through a combination of visual, somatosensory (haptic), and auditory feedback makes it an ideal tool for research in this field, as intelligent manipulation of these parameters can provide clinicians with an optimal but yet unattained level of control over the therapeutic efficacy of a given intervention [81].
3. Individual motivation: this variable favours neuroplasticity mechanisms [82], and it is considered essential to achieve an adequate compliance with therapy by the user. Motivation can be achieved by focusing the different activities that shape the therapy of the patient in a pleasant and attractive way. For example, it is known that when VR simulations are interconnected with motion capture systems, they provide a more engaging and motivating experience, as they get the user more involved in the therapy as the movement shown in the virtual world is a replica of the movement produced in the real world by the subject himself [83, 84].
4. Customization: VR-based therapies can be adapted to each patient by modifying parameters of the stimuli and the environment to provide an appropriate level of difficulty while maintaining attention and avoiding frustration or boredom. For example, contrast sensitivity, stimulus size, and spatial and temporal frequency, among others features, can be manipulated during amblyopia treatment. This is particularly relevant in dichoptic training, where visual stimuli can be adjusted to patients with strabismic amblyopia to facilitate the amblyopic eye perception and avoid suppression.

**Figure 1:**
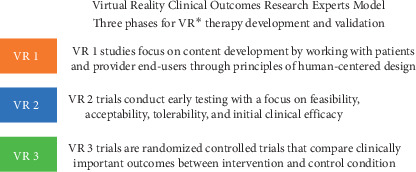
Summary of stages for the development and validation of VR-based therapies (https://www.ncbi.nlm.nih.gov/pmc/articles/PMC6374734/?report=classic).

In 2019, an international working group of 21 experts in the field, the Virtual Reality Clinical Outcomes Research Experts (VR-CORE) group, formally proposed a set of methodological guidelines to guide the design, implementation, analysis, interpretation, and communication of trials that develop and evaluate VR-based therapies. Using the FDA's Phase I-III pharmacotherapy model as guidance, committee members described various stages for the design and validation of VR-based therapies (Figure 1), beginning with content development in partnership with the end-users, progressing through initial clinical testing (usability and safety evaluation), and ending with properly powered and well-conducted RCTs [85]. The VR-CORE committee also outlined various considerations for each stage of the process, including the importance of standardizing control groups, selecting clinically relevant outcome measures, reporting which equipment was used in the trial, accounting for dropouts and disqualified participants, and finally allowing for pragmatic features of each study design.

## 5. Conclusions

VR technology can be used as a rehabilitation tool to exploit the sensory-motor adaptive capacity of the nervous system to compensate the deficits given in different pathologies, providing a technological method for intensive and repetitive training. This therapy approach also allows researchers to manipulate the specificity and frequency of sensory feedback provided to the patient, resulting in adaptive learning algorithms and graduated rehabilitation activities that can be objectively and systematically modified to create individualized treatment paradigms.

In recent years, more and more studies are emerging to test VR-based therapies efficacy in rehabilitation of different disorders; however, the effectiveness of these studies has not yet reached the higher levels of evidence found in large scale RCTs. Thus, more research is needed to establish the efficacy of sensory-motor rehabilitation through VR in various and larger clinical populations, and more importantly, to identify VR training parameters associated with an optimal transfer into real-world functional improvements.

In the case of amblyopia, few studies have explored the possibility of integrating perceptual learning and dichoptic stimulation approaches, which have a long history of suggesting effectiveness in improving visual skills, into VR environments.

In this context, results obtained from this line of research are positive, but there is still an important lack of well-designed studies suggesting a sustained effect on vision outside the intervention period. Likewise, the full potential of VR-based interventions will only emerge after we gain a deep understanding of how various sensory and haptic manipulations affect neural processes, so imaging studies to evaluate the effects of VR-based interventions on brain activation patterns and of various training parameters on long-term changes in brain function are needed to guide future clinical inquiry.

## Figures and Tables

**Table 1:** Summary of the clinical studies conducted to this date to evaluate the use of virtual reality technologies for the treatment of amblyopia.

Author (year)	Patients/groups	Age	Amblyopia type	Technology	Results	Follow-up
Waddingham et al. (2006) [64]	7 children	3 to 7 years	Residual amblyopia (anisometropic or strabismic with previous failed treatments)	I-BiT system (games and movie clips) Dichoptic	VA improvement from 6/12–6/120 pretreatment to 6/7.5–6/24–1 posttreatment Total treatment time 4.4 hours VA improvement in 5 out of 7 patients	7 to 15 sessions

Cleary et al. (2009) [65]	12 children	6.1 to 11.4 years	5 strabismic or mixed 7 anisometropic-strabismic	I-BiT system (driving game and video clip) Dichoptic 8 sessions, 25 min/session	58% sustained improvements in HCVA 67% improvement in LCVA Amblyopia eliminated in 2 patients 5 children with VA 6/12 or better at 6 months after stopping treatment HCVA improvement: 4 sessions LCVA improvement: 7 sessions	18 weeks

Herbison et al. (2013) [66]	10 children	Mean 5.4 years	3 strabismic, 4 anisometropic and 3 mixed	I-BiT system (Nux game and video clip) Dichoptic 6 weekly sessions, 30 min/session	78% showed VA improvement 67% demonstrated a clinically significant increase in VA of ≥0.125 (0.175 to 0.300 LogMAR) Mean change from baseline to follow-up was 0.13 ± 0.14 logMAR	10 weeks

Herbison et al. (2016) [68]	75 children	4 to 8 years	67 were residual amblyopes and 70 had an associated strabismus	Randomised control trial with three arms: (i) DVD footage shown to the amblyopic eye and common background to both (I-BiT DVD) (ii) Modified shooter game, Nux, with sprite and targets presented to the amblyopic eye (I-BiT game) (iii) Both background and foreground presented to both eyes (non-I-BiT) games)	VA improvement in all three arms by approx. 0.07 logMAR No difference between I-BiT DVD and non-I-BiT games compared with I-BiT games in terms of gain in vision	10 weeks

Cepeda-Zapata et al. (2019) [69]	45 students	17 to 28 years	Nonamblyopes	Conventional visual therapies implemented in virtual reality: brock cord, approach technique, and eccentric circles	Evaluation of simplicity and versatility for both clinician and infant patients Pragmatic quality above average (1.73) Attractiveness (2.03) and hedonic qualities (1.90) above average	N/A

Ziak et al. (2017) [70]	17 adults	17 to 69 years	Anisometropic	Diplopia game + Oculus Rift OC DK2 head mounted display 8 sessions, 40 min/session, twice per week Dichoptic	Mean BCVA in amblyopic eye improved from 0.58 ± 0.35 before training to a posttraining value of 0.43 ± 0.38 (*p* < 0.01) Mean stereoacuity changed from 263.3 ± 135.1” before dichoptic training to a value of 176.7 ± 152.4” after training *p* < 0.01 47.1% before dichoptic treatment had unmeasurable stereoacuity while this only occurred in 11.8% after training	1 month

^*∗*^VA, visual acuity; HCVA, high-contrast visual acuity; LCVA, low-contrast visual acuity.
